# Adult mortality in the cities of Bulawayo and Harare, Zimbabwe: 1979-2008

**DOI:** 10.1186/1758-2652-14-S1-S2

**Published:** 2011-07-06

**Authors:** Riitta A Dlodlo, Paula I Fujiwara, Zanele E Hwalima, Stanley Mungofa, Anthony D Harries

**Affiliations:** 1 International Union Against Tuberculosis and Lung Disease, 68 Boulevard St Michel, 75006 Paris, France; 2 Health Services Department, City of Bulawayo, PO Box 1946, Bulawayo, Zimbabwe; 3 Health Services Department, City of Harare, PO Box 596, Harare, Zimbabwe; 4 Department of Infectious and Tropical Diseases, London School of Hygiene and Tropical Medicine, Keppel Street, London, WC1E 7HT, UK

## Abstract

**Background:**

Zimbabwe has been severely affected by the HIV/AIDS and tuberculosis epidemics, with an estimated 80% of tuberculosis patients being HIV infected. We set out to use annual population-mortality records from the cities of Harare and Bulawayo to describe trends and possible causes of mortality from 1979 to 2008. The specific objectives were to document overall, sex and age-specific mortality, proportion of deaths attributed to AIDS and tuberculosis, and changes in death rates since the start of antiretroviral therapy in 2004.

**Methods:**

This retrospective descriptive study used existing mortality records of the Health Services departments in Harare and Bulawayo. Data points included: estimated yearly total population; groupings by sex and age; deaths (total and by sex and age groups for each year of the study period); and most frequently reported causes of death (for age groups <15 years, 15-44 years and ≥45 years). Data on deaths were aggregated by year, and crude, sex- and age-specific death rates were calculated per 1000 population. Tuberculosis and HIV-related disease-specific death rates and proportion of deaths attributed to these conditions were computed.

**Results:**

In both cities, crude death rates were lowest in the late 1980s, increased three- to five-fold by the early 2000s, and began a slow and, in the case of Bulawayo, intermittent decline from 2004. Sex-specific death rates followed a similar trend, being higher in males than in females. The death rates in the age groups <5 years, 15-44 years and ≥45 years showed significant increases, with a gradual levelling off and decline from 2002 onwards; death rates in those aged 5-14 years were relatively unaffected. Tuberculosis and HIV caused 70% of deaths in the age group of 15-44 years from the early 1990s.

**Conclusions:**

This study used routinely collected population-mortality data that are rare in resource-limited settings, and it described, for the first time in Zimbabwe, the effects of the HIV/AIDS epidemic and the introduction of antiretroviral therapy on death rates in two large cities. After a substantial rise in crude mortality rates, there has been a decline associated with the introduction of ART. Such routine population data must continue to be collected, collated and analyzed.

## Background

The HIV-1 epidemic became established in Zimbabwe in the mid-1980s and its subsequent spread was extremely rapid [1]. HIV prevalence peaked in 1997 when it was estimated to be 27% in the population aged 15 years and older [2]. Since the early 2000s, HIV prevalence has declined, with estimates of 24%, 18% and 14% in 2001, 2005 and 2009, respectively, in this age group [2]. This decline has been attributed to behaviour change and the impact of mortality [2].

HIV screening of all blood products started in the late 1980s. Voluntary counselling and testing and management of opportunistic infections became available in Zimbabwe in the mid-1990s, along with awareness campaigns, workplace and other peer education programmes, and social marketing and distribution of condoms. This was followed by services for prevention of mother to child transmission. From 2004-2005, provider-initiated testing and counselling for HIV was introduced at health facilities, particularly for patients with suspected or confirmed tuberculosis, when mass media crusades against concurrent partnerships were also initiated. The first five facilities to offer antiretroviral treatment (ART) in the public health sector opened in April 2004 and of these, three were situated in the cities of Harare and Bulawayo.

At the end of December 2009, of the 1.2 million individuals living with HIV, 219,000 (of whom 22,000 were children) had been started on ART [2]. ART coverage was estimated at 56%, based on the Guidelines for Antiretroviral Therapy in Zimbabwe [3] that recommended treatment for HIV-infected individuals with CD4 counts of <200 cells/mm^3^.

The first death from HIV infection in Zimbabwe was reported in 1988 in Harare. Since then, the country has seen a huge increase in HIV-related TB deaths, although there is almost no published data available on population mortality and its causes [4]. However, mortality statistics, collected and collated annually, have been available in bound volumes in the city councils of Harare and Bulawayo for several decades. They are believed to be fairly reliable for reasons described later.

The aim of the current study was to use these city records to describe the trends and possible causes of adult mortality over a 30-year period in Harare and Bulawayo. The specific objectives were to document by city: (i) overall mortality, including sex- and age-specific (15-44 years) mortality, from 1979 to 2008; (ii) the proportion of deaths attributed to AIDS and tuberculosis; and (iii) whether there was any reversal in death rates since the start of provision of ART.

## Methods

### Study design

This was a retrospective descriptive study using yearly mortality records in Bulawayo and Harare from 1979 to 2008.

### Study setting

#### General

In Zimbabwe, municipal authorities are responsible for provision of primary healthcare services and basic diagnosis and management of common conditions. Large municipalities, such as Harare and Bulawayo, have Health Services departments to carry out these duties. The study was conducted using annual reports from the Health Services departments of Harare and Bulawayo. The population estimates of the two cities were based on the national census reports of 1982, 1992 and 2002, with interim numbers provided by the projections of the Central Statistical office.

HIV prevalence in the two cities has been high since the start of the HIV/AIDS epidemic, and in 2009, it was found to be 12% in Harare and 14% in Bulawayo among antenatal care clients aged 15 to 49 years [5]. By the end of 2008, there were five public health sector ART-initiating sites in Harare and six in Bulawayo. The number of individuals ever started on ART in the Bulawayo sites, including four primary healthcare clinics, was approximately 30,000 by 31 May 2010, which was estimated to represent 45% of the need. These estimates were unavailable for Harare.

#### Annual mortality reports

Annual reports are prepared by the Health Services departments and presented to city councillors and shared with relevant stakeholders. These reports have been available for several decades in Bulawayo and Harare. Though the reporting styles are not identical and have changed, especially after 1980 when the country gained its independence, most reports include a section on vital events, such as deaths, that are frequently disaggregated by sex and age group. Mortality data include deaths that occur both in health facilities and homes (the latter accounting for up to 40% of all deaths [unpublished data]). These data are believed to be fairly comprehensive because no burial in city cemeteries that are managed by the Health Services departments is permitted without registration of a death. Transportation of a body out of the city without necessary documentation is also impossible as it is reinforced by police officers who man roadblocks along the highways.

A death notification is issued by a medical officer at a facility where the death occurred or where the deceased received care. This form captures the direct, underlying and contributing causes of death, and allows the acquisition of an internment order (issued by the Health Services departments) and a burial order and death certificate (issued by the Registrar for Births and Deaths).

The departmental clerks collect information from the Registrar’s offices on the number of deaths that have occurred among residents in the city during the year. Clerks also prepare line listings that consist of age, sex and causes of death as written in death certificates, and exclude personal identifiers, such as name and address of the deceased. These manual lists are then examined by medical officers who code the causes of death, if more than one has been included in the certificate, into broad categories, such as tuberculosis, AIDS, pneumonia/chest infections, and accidents. The information is then summarized in tables and presented in the departmental annual reports.

### Data collection

The annual reports from each city from 1979 to 2008 were retrieved and data on deaths were extracted. The yearly population estimates that the departments had used were also summarized. A spread-sheet template was developed to enter: total population and population by sex and age group; total number of deaths and number of deaths by sex and age groups (less than five years, 5-14 years, 15-44 years and 45 years and above); and the most frequently reported causes of death (for age groups younger than 15 years, 15-44 years, and 45 years and above).

### Analysis

Data on deaths, including deaths stratified by sex and age, were aggregated by year and the crude, sex- and age-specific death rates were calculated per 1000 population based on population estimates for each city. Chi-square for trend test was performed on the yearly reduction in the crude mortality rate from 2003-2004.

For cause-specific mortality, we focused on tuberculosis and HIV-related disease for the age group of 15-44 years and from 1988 to 2008 only. We included all types of tuberculosis, and the proportion of deaths attributed to tuberculosis was calculated for each year. For HIV-related disease, we included death due to HIV-related disease, tuberculosis, pneumonia/*Pneumocystis jirovecii* (or previously, *carinii*) pneumonia, meningitis, malnutrition, Kaposi’s sarcoma and septicaemia; the proportion of deaths attributed to HIV-related disease was calculated for each year. Deaths due to tuberculosis and to HIV/ AIDS are, therefore, not mutually exclusive in this analysis.

### Ethics approval

Permission for the study was obtained from the Directors of the two Health Services departments and the Ethics Advisory Group of the International Union Against Tuberculosis and Lung Disease.

## Results

### Population

The estimated total population in Harare increased from 653,800 in 1980 to 1,532,800 in 2008 [6 and unpublished data]*.* In 2008, 765,000 (50%) inhabitants were females. The estimated total population in Bulawayo increased from 391,100 in 1980 to 719,500 in 2008 [6 and unpublished data]*.* In 2008, there were 369,500 (51%) females.

### Crude death rate

The total number of deaths during the 30-year period was 283,926 in Harare and 200,124 in Bulawayo. The trends in crude death rates were similar in both cities, with Bulawayo having consistently higher rates than Harare.

**Figure 1:**
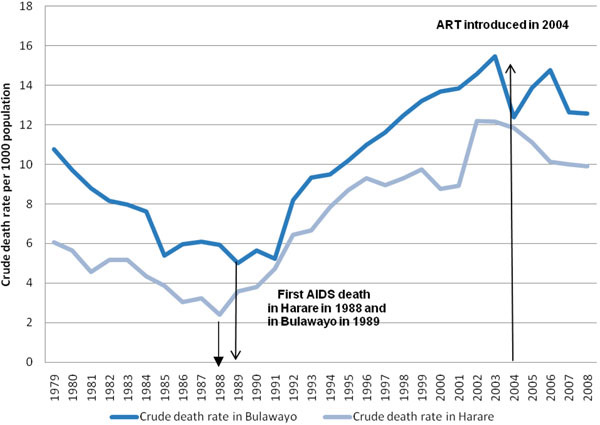
Crude death rate in Bulawayo and Harare from 1979 to 2008.

The crude death rate per 1000 population was 6.1 in Harare and 10.8 in Bulawayo in 1979 (Figure 1). The rate declined over the next decade in both cities, reaching its nadir in Harare in 1988 at 2.4 per 1000 population and in Bulawayo in 1989 at 5.0 per 1000 population. Thereafter, the rates increased annually and peaked in 2002-2003 at 12.2 and 15.5 per 1000 population in Harare and Bulawayo, respectively. From 2003-2004, the crude death rate showed a steady decline in Harare and an intermittent decline in Bulawayo. In 2008, the rates were 9.9 and 12.6 per 1000 population in Harare and Bulawayo, respectively. Overall, there was a 19% decrease in crude death rates in both cities between 2003 and 2008: for Harare, the chi-squared test for trend was 629.8 (p <0.001) and for Bulawayo, it was 121.4 (p <0.001).

### Sex-specific death rate

**Figure 2:**
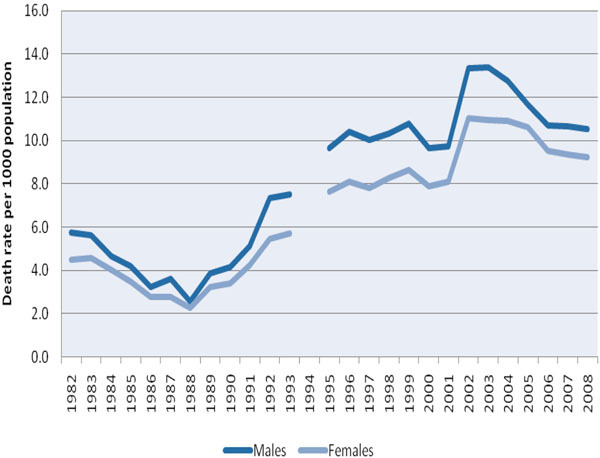
Sex-specific death rate in Harare from 1982 to 2008.

In both cities, there were some years when mortality information by sex, age group and cause was unavailable (Table 1). In Harare, sex-specific death rates were available for a 26-year period (1982-1993 and 1995-2008), and these rates are shown in Figure 2. During all years, mortality in males was higher than in females. The overall trend of the sex-specific death rates resembled the trend seen in crude death rates in that the lowest rates were recorded in the late 1980s (when the death rate was 2.3 and 2.5 per 1000 population in females and males, respectively) and the highest in the early 2000s (when the death rate was 11.1 and 13.3 per 1000 population in females and males, respectively), after which the rates started to decline.

**Table 1:** Summary of available death-related information in the departmental annual reports in Bulawayo and Harare from 1979 to 200

Type of information	Years when available in annual reports in Bulawayo	Years when available in annual reports in Harare
Total number of deaths	From 1979 to 2008 (30 years)	From 1979 to 2008 (30 years)
Number of deaths by sex	1985-1989, 1991, 1993-1994, 1998-2003, 2005-2008 (18 years)	1982-1993, 1995-2008 (26 years)
Number of deaths by age group	1986-1989, 1991, 1993-1994, 1996, 1998-2008 (18 years)	1979-2008 (30 years for < 5 and ≥ 45) 1980-2008 (29 years for 5-14 and 15-44 )
Causes of death for 15-44 age group	1979-1989, 1991, 1993-94, 1996, 1999-2008 (25 years)	1981, 1983, 1985, 1987-2008 (25 years)

There were more missing data in Bulawayo, but these (not shown) followed the same trends as were seen in Harare, although death rates were consistently higher than in Harare, especially among males, in whom a mortality rate of 17.8 per 1000 population was reported in 2003.

### Age-specific death rate

**Figure 3:**
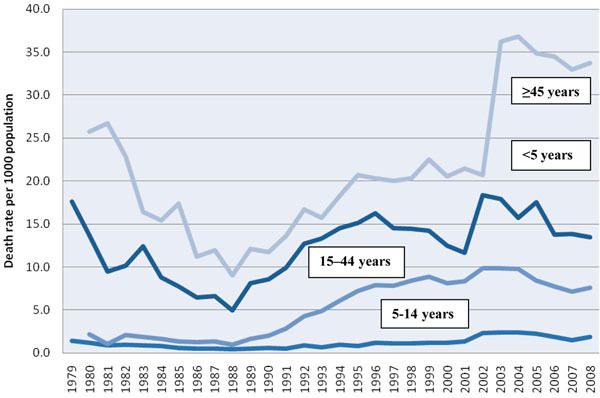
Age-specific death rate in Harare from 1979 to 2008.

In Bulawayo, there were several years when information on age-specific death rates was unavailable (Table 1). However, in Harare, these data were available for a 29-year period from 1980 to 2008 for the age groups of 5-14 and 15-44 years, and for a 30-year period for the other age groups (Figure 3).

In all age groups, the lowest death rates during the study period were recorded from the mid-1980s to 1988 and thereafter, the mortality rose, peaked at 9.9 per 1000 population in the age group of 15-44 years from 2002 to 2004, and subsequently declined. Three age groups were principally affected during these 30 years (younger than five years, 15-44 years, and 45 years and above), with all three showing an increase in mortality from 1989 to 2002, and thereafter, a decline reaching 7.6 per 1000 population (15-44 years) in 2008. The age group of 5-14 years was relatively unaffected.

In Bulawayo, all-cause mortality in the age group of 15-44 years followed a similar trend and reached its highest point in 2003 at 15.7 per 1000 population and then declined to 12.3 per 1000 population in 2008 (not shown). Similar to Harare, the three age groups predominantly affected were younger than five years, 15-44 years, and 45 years and above, while the age group of 5-14 years was relatively unaffected.

### Cause-specific mortality in the 15-44 year age group

**Figure 4:**
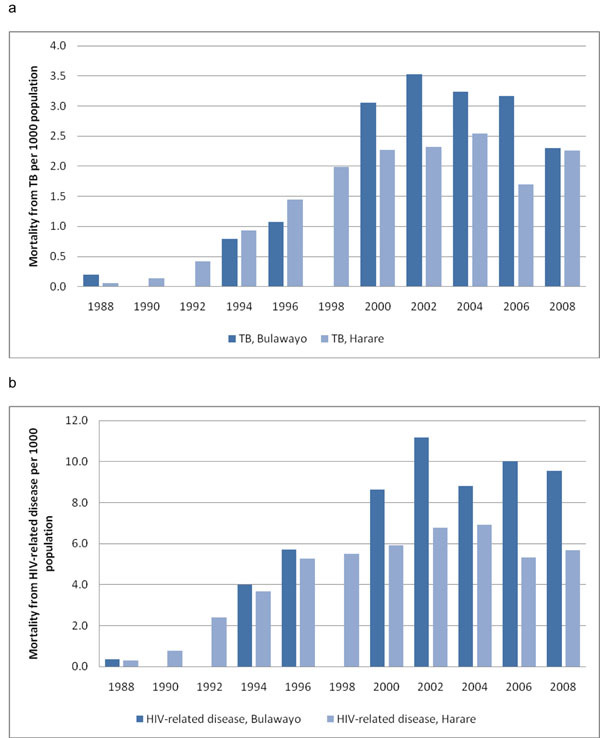
(a) Mortality from tuberculosis in Bulawayo and Harare from 1988 to 2008. (b) Mortality from HIV-related disease in Bulawayo and Harare from 1988 to 2008.

Cause-specific mortality due to tuberculosis and HIV-related disease in the age group of 15-44 years from 1988 to 2008 is shown in Figures 4a and 4b. Data were unavailable in Bulawayo in 1990, 1992 and 1998. TB-specific mortality increased 18-fold in Bulawayo and 25-fold in Harare, and was 2.3 per 1000 population in both cities in 2008. Mortality due to HIV-related disease increased 23-to 28-fold, and the death rate was 5.7 and 9.5 per 1000 population in Harare and Bulawayo, respectively, in 2008.

### Proportionate mortality ratio in the 15-44 year age group

**Figure 5:**
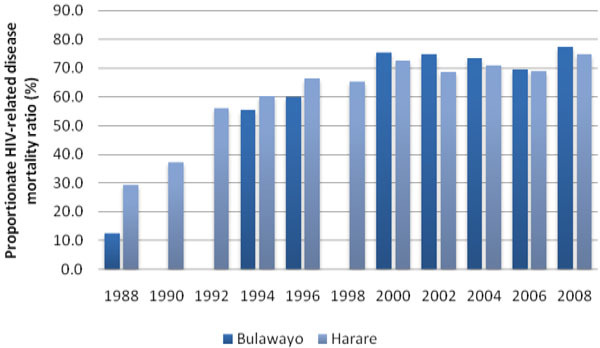
Proportionate mortality ratio due to HIV-related disease, including tuberculosis, in Bulawayo and Harare from 1988 to 2008.

The proportion of all deaths in this age group that were recorded as due to HIV/AIDS and related conditions, as stated in the Methods section, is shown in Figure 5. Data were unavailable in Bulawayo in 1990, 1992 and 1998. Initially low, the proportion increased to more than 70% in 2000 and has remained at that level ever since.

## Discussion

This is the first study in Zimbabwe to use routinely collected mortality data to assess the impact of the HIV/ AIDS epidemic and introduction of antiretroviral therapy on death rates in Harare and Bulawayo. Crude death rates in both cities rose staggeringly between the late 1980s and early 2000s and began a slow and, in the case of Bulawayo, intermittent decline from 2003-2004, coincident with the advent of antiretroviral therapy. Interestingly, during the entire study period, overall mortality was consistently higher in Bulawayo than in Harare, and the reason for this difference is not known. Sex-specific death rates followed a similar trend, being higher in males than in females.

Not surprisingly, the death rates in the age groups younger than five years, 15-44 years, and 45 years and above showed significant increases during the same period and a gradual levelling off and decline from 2002-2003 onwards. The age group relatively unaffected during this time was that of persons aged 5-14 years, who epidemiologically would have been protected against the ravages of the HIV/AIDS epidemic. Finally, there was an enormous rise in tuberculosis- and HIV-specific mortality in the age group of 15-44 years from 1988 to 2008, and from the early 1990s, the majority of adult deaths were due to HIV/AIDS and tuberculosis.

The strengths of this study relate to the collection and compilation of death information in both cities by the Health Services departments, a system that has continued uninterrupted over the years despite major political and economic challenges faced by the country during some of this time. We believe that the numerical data on deaths in urban areas are fairly reliable because no burial in a city cemetery or transportation of a body out of the city could and can be done without documentation. This differs significantly from death registration coverage in rural areas, where many deaths may go unreported [1]. The data also fit with what is known about the epidemiology of the HIV infection, especially when disaggregated by age group.

However, this was an operational study based on data collected within routine systems for which there are obvious limitations. We do not know the accuracy of the annual population estimates for the cities based on the 10-year national census data with projected numbers in between. These data may be affected by urban-rural and rural-urban migration, as well as by emigration during recent years of insecurity and economic turmoil. The population denominator data may, therefore, have in-accuracies which will affect the results.

While we believe the number of annual deaths to be fairly accurate, this may not be the case. The Registrar’s office should record all deaths that occur in the city for that year, whether the person is resident or not in the city, but deaths in infants and children in particular may not always have been captured. Over the 30-year period, there may have been changes in health information clerks, who may have used different criteria for inclusion and exclusion of deaths. There were also no standard operating procedures for recording deaths, so practices between medical officers in different cities and within a city over time might have differed, creating inaccurate attributions regarding causes of death. The format of the death notification requires determination of direct, underlying and contributing causes of death, although frequently, in practice, the cause of death is limited to just one line, for example, “retroviral infection”. Although the International Classification of Diseases and Deaths (ICD) is recognized in Zimbabwe, most causes of death are not accompanied by an ICD-recommended code.

Autopsy is the only way to truly ascertain cause of death, but in Zimbabwe, as is the case in most of sub-Saharan Africa, they are rarely performed. Many deaths also occur at home, especially in the case of chronic illness due to HIV/AIDS and TB, where medical certification might have been cursory, and this may have lead to an over-estimation of deaths attributed to HIV/AIDS and TB. Finally, it would have been interesting to assess the effect of the HIV/AIDS epidemic on non-HIV related deaths and broader health outcomes, but the ways in which the annual reports present the data precluded such analysis.

Our results are supported by previous studies in a Zimbabwean province, Manicaland, which also described increased mortality among young and middle aged adults, with 61% and 70% of mortality in males and females, respectively, being attributed to HIV/AIDS [1,7]. Modelling studies also strongly suggest that the increased mortality rates were due to HIV/AIDS [8]. The Zimbabwe Demographic and Health Surveys [9] found higher death rates for males than females in 1994, 1999 and 2005, but mortality rates were not shown by province and therefore, no data were available for Harare and Bulawayo.

There have been a few population-based studies in other African countries assessing the impact of HIV/ AIDS and the advent of antiretroviral therapy on population mortality. Studies have been conducted in Ethiopia [10,11], Botswana [12], South Africa [13, 14, 15] and Malawi [16,17] assessing the impact of antiretroviral therapy on HIV-related deaths at the population level, and showing that the numbers of HIV/AIDS-related deaths have declined substantially with ART. The reductions in death rates in Africa have generally been less dramatic than those reported from New York [18] and São Paulo [19].

In our study, the decline in the crude death rate was only 19%, which may be due to several factors. First, the HIV incidence in Harare and Bulawayo appeared to have peaked early in relation to ART: back-calculation from mortality data indicated that an annual incidence peak of 4-5% could have been reached in Harare between 1988 and 1990. In Bulawayo, a higher annual incidence peak of 5-6% was estimated to have occurred between1992-1994 [20]. Second, the number of HIV and AIDS cases was and continues to be large, with consequent poor coverage of ART as a result of the immense work load and relatively weak health infrastructure. Third, socio-cultural and stigma-related factors frequently impede demand for HIV testing and make persons present late for HIV diagnosis and treatment, which compromises survival [17].

Interestingly, studies from Manicaland, before availability of ART, reported stabilization of mortality rates because of decreasing HIV prevalence partly due to the natural consequence of the HIV epidemic and partly due to recent behavioural changes as a result of HIV prevention programmes in the country [21-25]. Our data from urban Zimbabwe add to this growing literature from the African continent, support the general impression of a decline in population death rates consequent upon expansion of antiretroviral treatment, and provide a 30-year glimpse at mortality trends in two large African cities that span the pre-AIDS era, the HIV/AIDS epidemic and the advent of ART.

More importantly, this study also demonstrates the importance and value of well-kept records. Routine systems that can measure population numbers and population deaths on an annual basis are rare in resource-poor countries [26], but where they do exist, as in Harare and Bulawayo, they need to be used, supported and developed so that the data can become more reliable and useful as a measure of what is happening at the population level. Routine usage and regular supervision will help improve the reliability of the data, and the value of such data needs to be demonstrated to policy makers so that commitment towards collection, collation and analysis is attained.

## Conclusions

It is important to collect evidence to show that efforts to provide HIV diagnosis and care, including ART, and funds invested by both national governments and various funding agencies have an impact in averting premature deaths [27,28]. Furthermore, two Millennium Development Goals have mortality-based targets, and evidence is also needed to assess whether these targets are being reached.

This study used routinely collected mortality data that are rare in resource-limited settings, and described, for the first time in Zimbabwe, the effects of the HIV/AIDS epidemic and introduction of ART on death rates in two large cities. It shows the value of these data in describing the effect of the HIV/AIDS epidemic and the impact of HIV care and ART on population-level mortality in spite of certain limitations, such as unknown accuracy of population estimates in the cities and lack of autopsy-confirmed causes of death. The data can also be used in the future to assess population-level impact of better access to care through expansion and decentralization of HIV treatment services.

## Competing interests

The authors declare that they have no competing interests.

## Authors’ contributions

RAD and ADH conceived the idea of this study and designed the tool to be used. RAD carried out the data collection with the research assistants that she inducted and supported, validated and corrected data entry, analyzed the data and wrote the first draft of the manuscript, which ADH reviewed extensively. PIF, ZEH and SM reviewed the manuscript critically.
